# The evolving story of *Borrelia burgdorferi* sensu lato transmission in Europe

**DOI:** 10.1007/s00436-022-07445-3

**Published:** 2022-02-05

**Authors:** Antje Steinbrink, Katharina Brugger, Gabriele Margos, Peter Kraiczy, Sven Klimpel

**Affiliations:** 1 Institute for Insect Biotechnology, Justus-Liebig-University of Giessen, Heinrich-Buff-Ring 26, 35392 Giessen, Germany; 2 LOEWE Centre for Translational Biodiversity Genomics (LOEWE-TBG), Senckenberganlage 25, 60325 Frankfurt am Main, Germany; 3 Unit for Veterinary Public Health and Epidemiology, University of Veterinary Medicine Vienna, Veterinärplatz 1, 1210 Vienna, Austria; 4 German National Reference Centre for Borrelia, Bavarian Health and Food Safety Authority Branch, Veterinärstraße 2, 85764 Oberschleißheim, Germany; 5 Institute of Medical Microbiology and Infection Control, University Hospital of Frankfurt, Goethe University Frankfurt, Paul-Ehrlich-Straße 40, 60596 Frankfurt am Main, Germany; 6 Institute of Ecology, Evolution and Diversity, Goethe University Frankfurt, Max-von-Laue-Straße 13, 60438 Frankfurt am Main, Germany; 7 Senckenberg Biodiversity and Climate Research Centre, Senckenberg Gesellschaft für Naturforschung, Senckenberganlage 14, 30325 Frankfurt am Main, Germany

**Keywords:** *Borrelia*, *Ixodes*, Lyme borreliosis, Lyme disease, Tick, Tick-borne diseases, Spirochetes

## Abstract

Beside mosquitoes, ticks are well-known vectors of different human pathogens. In the Northern Hemisphere, Lyme borreliosis (Eurasia, LB) or Lyme disease (North America, LD) is the most commonly occurring vector-borne infectious disease caused by bacteria of the genus *Borrelia* which are transmitted by hard ticks of the genus *Ixodes*. The reported incidence of LB in Europe is about 22.6 cases per 100,000 inhabitants annually with a broad range depending on the geographical area analyzed. However, the epidemiological data are largely incomplete, because LB is not notifiable in all European countries. Furthermore, not only differ reporting procedures between countries, there is also variation in case definitions and diagnostic procedures. Lyme borreliosis is caused by several species of the *Borrelia (B.) burgdorferi* sensu lato (s.l.) complex which are maintained in complex networks including ixodid ticks and different reservoir hosts. Vector and host influence each other and are affected by multiple factors including climate that have a major impact on their habitats and ecology. To classify factors that influence the risk of transmission of *B. burgdorferi* s.l. to their different vertebrate hosts as well as to humans, we briefly summarize the current knowledge about the pathogens including their astonishing ability to overcome various host immune responses, regarding the main vector in Europe *Ixodes ricinus*, and the disease caused by borreliae. The research shows, that a higher standardization of case definition, diagnostic procedures, and standardized, long-term surveillance systems across Europe is necessary to improve clinical and epidemiological data.

## Introduction

Vector-borne diseases (VBD) are a major global public health threat that affect more than one billion people and account for more than 700.000 deaths annually (WHO 2020). Most of the VBD are reported by low-income countries in tropical and subtropical regions, but also countries of the Northern Hemisphere are faced with ongoing infections caused by vector-borne pathogens (WHO 2020). The most commonly occurring VBD of the Northern Hemisphere is Lyme borreliosis (LB) in Eurasia or Lyme disease (LD) in North America. Several species of the so-called *Borrelia* (*B*.) *burgdorferi* sensu lato (s.l.) complex are the causative agents of LB, all of which are transmitted by hard ticks of the genus *Ixodes* (ECDC 2014; CDC 2021)*.* In Europe, the reported incidence of LB cases per 100,000 inhabitants ranged from 0.6 in Ireland, to 80 in Sweden, and to 300 in Austria (Lindgren and Jaenson 2006). There are approximately 85,000 cases of LB notified in Europe each year (Sykes and Makiello 2017). In Germany, 60,000–200,000 cases have been estimated annually but the “true” number of LB cases lies likely somewhere in between (Müller et al. 2012; Hofmann et al. 2017; Rauer et al. 2020). Apparently, there is considerable epidemiological uncertainty regarding LB incidence and prevalence in Europe (Lindgren and Jaenson 2006; Hofmann et al. 2017; Rauer et al. 2020). Not only varies the annual incidence of reported LB cases greatly across European countries, there are also differences in the implemented surveillance systems in individual countries. This might account for the variation in the incidences as some take into consideration clinically well-diagnosed cases, while others also report probable or suspected LB cases. Furthermore, inconsistent case definitions, lacking of an implemented quality management system and non-uniform diagnostic procedures contribute to inaccurate epidemiological data (Lindgren and Jaenson 2006; Müller et al. 2012; Wilking and Stark 2014; Enkelmann et al. 2018).

Thus, observed increases in incidence or annual cases might be due to different data sources, changes in reporting procedures, expansion of ticks into regions previously cleared of ticks, or into newly created habitats, e.g., by renaturation and development of new environments, dispersal of natural hosts into low incidence regions, or due to increased awareness of general practitioners and the lay public (Kugeler et al. 2021). As *Borrelia* are strictly dependent on their vectors and reservoir hosts, it is important to understand the biotic and abiotic factors that determine the ecological dynamics in tick and *Borrelia* spreading to discern and manage the risk of humans to acquire the agent and develop LB. There is clearly a need to study these complex ecological networks over long periods of time, especially in the light of climate change, as tick life cycles can take several years to be accomplished and reservoir hosts like birds, squirrels, or hedgehogs are long-lived vertebrates (Hartemink et al. 2015; Stone et al. 2017). In this review, we give a brief overview about the driving factors making *B. burgdorferi* s.l. a human pathogen in Europe and where we stand and what should be done in the future for a better understanding of the close relationships between the pathogen, vector, hosts, and humans. Such knowledge can be used to enhance public information, to sensitize decision-makers and to improve diagnostic standards to prevent late manifestations.

## The *B. burgdorferi* s.l. species complex

**Figure 1:**
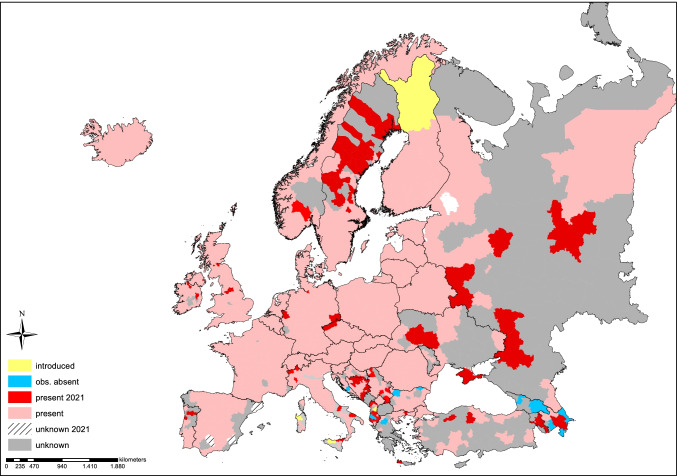
Distribution and changes in the prevalence of *Ixodes ricinus*, the main vector of the *Borrelia burgdorferi* s.l. complex among Europe. The map is a compilation of reported findings of *I. ricinus* in 2017 (rosé and light blue) in comparison to 2021 (others color codes) reported to the ECDC (European Centre for disease Prevention and Control). Please note that the map depicts historical and actual findings condensed on NUTS-3 level, the European socio-economic, or regional administrative level. However, no distinction is made between individual findings or stable populations. In addition, areas with “no data” should not interpreted as whether the species does or does not exist. Original maps can be accessed online: https://www.ecdc.europa.eu/en/disease-vectors/surveillance-and-disease-data/mosquito-maps. Map was created with ArcGIS 10.8.

**Figure 2:**
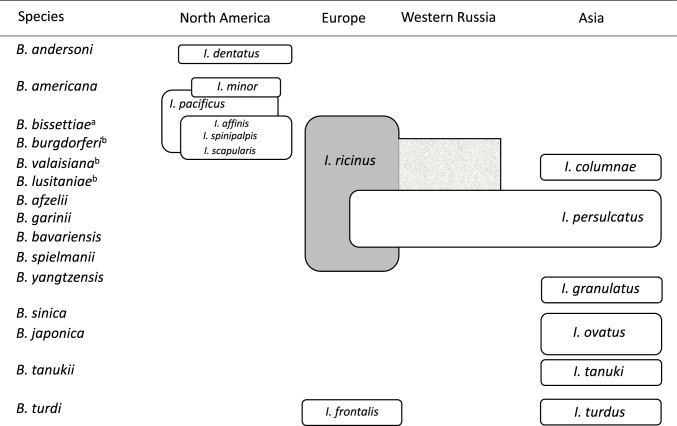
Geographic distribution and vector associations of *Borrelia burgdorferi* s.l. adapted after Margos et al. (2012) with new included *Ixodes frontalis* as vector for *B. turdi* in Europe. Abbreviations: I., *Ixodes*; B. *Borrelia*; ^a^for *B. bisettiae*, the vector for Europe is uncertain; ^b^*B. burgdorferi*, *B. valaisiana*, and *B. lusitaniae* are transmitted by *I. ricinus* in Eastern Europe but their prevalence is low.

Spirochetes which comprise the *B. burgdorferi* s.l. complex were first discovered in ixodid ticks in the early 1980s (Burgdorfer et al. 1982) although it had been suspected since the beginning of the last century that tick-borne pathogens may cause symptoms that are now known as LB (reviewed by Stanek et al. 2002). The bacterium was named *B. burgdorferi* (Johnson et al. 1984), and it was believed to be a single bacterial species. The diversity of the species complex became apparent during subsequent investigations that unraveled the genetic and ecological heterogeneity of borreliae in Europe, Asia, and North America. Several new genospecies were identified in Europe but also in North America and Asia (Table 1) (Anderson et al. 1989; Baranton et al. 1992; Kawabata et al. 1993; Postic et al. 1993; Canica et al. 1993; Fukunaga et al. 1996; Le Fleche et al. 1997; Richter et al. 2006; Postic et al. 2007; Rudenko et al. 2009, 2011; Margos et al. 2013; Ivanova et al. 2014; Margos et al. 2014, 2015, 2016, 2017, 2020; Pritt et al. 2016). Since then, the name *B. burgdorferi* s.l. has been used to refer to the species complex, while *B. burgdorferi* s.s. refers to the species first discovered by Willy Burgdorfer and collaborators in the USA (Burgdorfer et al. 1982; Johnson et al. 1984). Currently, the *B. burgdorferi* s.l. species complex contains more than 20 validated and proposed genospecies (Table 1) of which six are assured human pathogens. The species are non-uniformly distributed mainly in the Northern Hemisphere between the latitudes of 40 and 60° N. This distribution also reflects the presence of ixodid ticks (see Figures 1 and 2) and reservoir hosts. The ecological systems supporting natural transmission cycles of *Borrelia* are highly complex, and competent reservoir hosts and vectors may occur in sympatry with hosts or ticks with reduced or no reservoir/vector competence. The latter will negatively affect the success of *Borrelia* species or individual strains (Tsao 2009; Margos et al. 2019). Furthermore, not all vertebrates that are infected with a *Borrelia* species do indeed serve as a reservoir host. For many animal species, experimental evidence on reservoir competence has not been established so far (Wolcott et al. 2021). A rough indication of animal taxa that may serve as reservoirs for the different *Borrelia* species although not all species within these taxa may be competent reservoir hosts is summarized in Table 1. Seemingly, many *Borrelia* species have narrow host preferences, and very few species can use divergent taxa as reservoir hosts. An example for a “generalist” species is *B. burgdorferi* s.s. as it can use diverse mammalian and avian hosts as reservoir (Hanincová et al. 2006).

**Table 1:** Members of the *Borrelia burgdorferi* s.l. complex with their year of definition and valid publication, reference of description and their geographical distribution, suspected reservoir hosts, suspected vector species, and influence on human health. Species distributed in Europe are indicated in bold. The species *B. finlandensis* proposed by Casjens et al. (2011) is not included in this table because the *Borrelia* isolate (SV1) which was used to “define” the species *B. finlandensis* clusters in MLST phylogenies in the same clade as isolates NE49 and Z41293, and these were used to define species borders, i.e., they are enclosed in *B. burgdorferi* s.s. (see Postic et al. 2007, Margos et al. 2009).

***Borrelia s***pecies	Type strain	Year of definition	Year of valid publication	Suspected reservoir hosts	Suspected vector ***Ixodes*** spp.	Distribution	Human pathogenicity
***B. afzelii***	VS461	1993 (Canica et al. 1993)	1994 (Validation list no. 48. Int J Syst Bacteriol 1994; 44:182-183)	Rodents, insectivores	*I. ricinus, I. persulcatus, I. hexagonus*	Asia, Europe	Yes
*B. americana*	SCW-41	2007 (Postic et al. 2007)	2009 (Rudenko et al. 2009)	Birds, rodents	*I. minor, I. pacificus*	North America	Unknown
*Candidatus B. andersonii*	21038	1995 (Marconi et al. 1995)		Birds, rabbits	*I. dentatus*	North America	Unknown
*Candidatus B. aligera*		2020 (Norte et al.2020b)		Unknown	Unknown	Unknown	Unknown
***B. bavariensis***	PBi	2009 (Margos et al. 2009)	2013 (Margos et al. 2013)	Rodents	*I. ricinus, I. persulcatus*	Asia, Europe	Yes
***B. bissettiae***	DN-127	1998 (Postic et al. 1998)	2016 (Margos et al. 2016)	Rodents	*I. spinipalpis, I. pacificus*	Europe, North America	Potentially
***B. burgdorferi* s. s.**	B31	1984 (Johnson et al. 1984)	1984 (Johnson et al. 1984)	Birds, rodents, insectivores, carnivores	*I. ricinus, I. scapularis, I. affinis, I. pacificus, I. minor, I. hexagonus*	Europe, North America	Yes
*B. californiensis*	CA446	2007 (Postic et al. 2007)	2016 (Margos et al. 2016)	Rodents	*I. pacificus, I. spinipalpis, I. jellisoni*	North America	Unknown
*B. carolinensis*	SCW-22	2011 (Rudenko et al. 2011)	2011 (Rudenko et al. 2011)	Rodents	*I. minor*	North America	Unknown
*B. chilensis* (p)	VA1 (p)	2014 (Ivanova et al. 2014)		Rodents	*I. stilesi*	South America	Unknown
***B. garinii***	20047	1992 (Baranton et al. 1992)	1992 (Baranton et al. 1992)	Birds	*I. ricinus, I. persulcatus, I. uriae*	Asia, Europe	Yes
*B. japonica*	HO14	1993 (Kawabata et al. 1993)	1994 (Validation list no. 50. Int J Syst Bacteriol 1994; 44:595)	Rodents	*I. ovatus*	Asia	Unknown
*B. kurtenbachii*	25015	2010 (Margos et al. 2010)	2014 (Margos et al. 2014)	Rodents	Unknown	North America	Potentially
*B. lanei*	CA28-91	2007 (Postic et al. 2007)	2017 (Margos et al. 2017)	Lagomorphs?	*I. spinipalpis, I. pacificus*	North America	Unknown
***B. lusitaniae***	PoTiB2	1997 (La Fleche et al. 1997)	1997 (La Fleche et al. 1997)	Lizards	*I. ricinus*	Europe	Potentially
*B. maritima*	CA690	2020 (Margos et al. 2020)	2020 (Margos et al. 2020)	Unknown	*Unknown*	North America	Unknown
*B. mayonii*	M14-1420	2016 (Pritt et al. 2016)	2016 (Pritt et al. 2016)	Rodents?	*I. scapularis*	North America	Yes
*B. sinica*	CMN3	2001 (Masuzawa et al. 2001)	2001 (Masuzawa et al. 2001)	Rodents	*I. ovatus*	Asia	Unknown
***B. spielmanii***	PC-Eq17	2004 (Richter et al. 2004)	2006 (Richter et al. 2006)	Rodents	*I. ricinus, I. hexagonus*	Europe	Yes
*B. tanukii*	Hk501	1996 (Fukunaga et al. 1996)	1996 (Fukunaga et al. 1996)	Rodents	*I. tanuki*	Asia	Unknown
***B. turdi***	Ya501	1996 (Fukunaga et al. 1996)	1996 (Fukunaga et al. 1996)	Birds	*I. turdus, I. frontalis, I. ricinus*	Asia, Europe	Unknown
***B. valaisiana***	VS116	1997 (Wang et al. 1997)	1997 (Wang et al. 1997)	Birds	*I. ricinus*	Europe	No
*B. yangtzensis*	Okinawa CW62	2008 (Chu et al. 2008)	2015 (Margos et al. 2015)	Rodents	*I. granulatus*	Asia	Potentially

## *Ixodes ricinus*: the European vector for *B. burgdorferi* s.l.

More than 700 hard ticks (Acari: Ixodida: Ixodidae) are described worldwide, around 38% of which are feeding on humans but only 3% regularly (Guglielmone et al. 2014; Cutler et al. 2021). Almost 13.2 % of the 700 tick species are native in the Palearctic region. They are highly efficient vectors for pathogens of humans, livestock, companion animals, and wildlife **(**Estrada-Peña et al. 2017). In Europe, the most common endemic tick species is *Ixodes ricinus*, which is also known as castor bean tick or—on the British Isles—sheep tick (Estrada-Peña et al. 2013; Rubel et al. 2021). It is probably the best-studied species regarding its biology, ecology, and role as a vector for certain pathogens (Gray et al. 2016). In the northeast, its distribution overlaps with another *B. burgdorferi* s.l. vector, *I. persulcatus* (Swanson et al. 2006). *Ixodes ricinus* prefers habitats that offer optimal microclimatic conditions and a host composition suitable for blood meals of all three life stages. Especially, a permanent, deep leaf litter provides microclimatic temperature and humidity conditions for all developmental tick stages that enable them to survive for the time between blood meals, even in temporarily dry periods (Kahl 2018). Hence, high tick densities are typically found in deciduous or mixed forest but also in coniferous forest, urban parks, cemeteries, or even gardens close to woods (Gray et al. 1998; Boehnke et al. 2015; Brugger et al. 2016). Also*, I. hexagonus* and *I. persulcatus* are proven vectors for *B. burgdorferi* s.l. (Eisen 2020). Although *B. burgdorferi* s.l. has frequently been detected in other hard-bodied tick species, e.g*.*, *Dermacentor reticulatus* and *Haemaphysalis concinna*, it was experimentally confirmed that they are not vector competent for *Borrelia* (Eisen and Lane 2002; Eisen 2020).

## The interrelationship between *B. burgdorferi* s.l., ticks, and their vertebrate hosts

**Figure 3:**
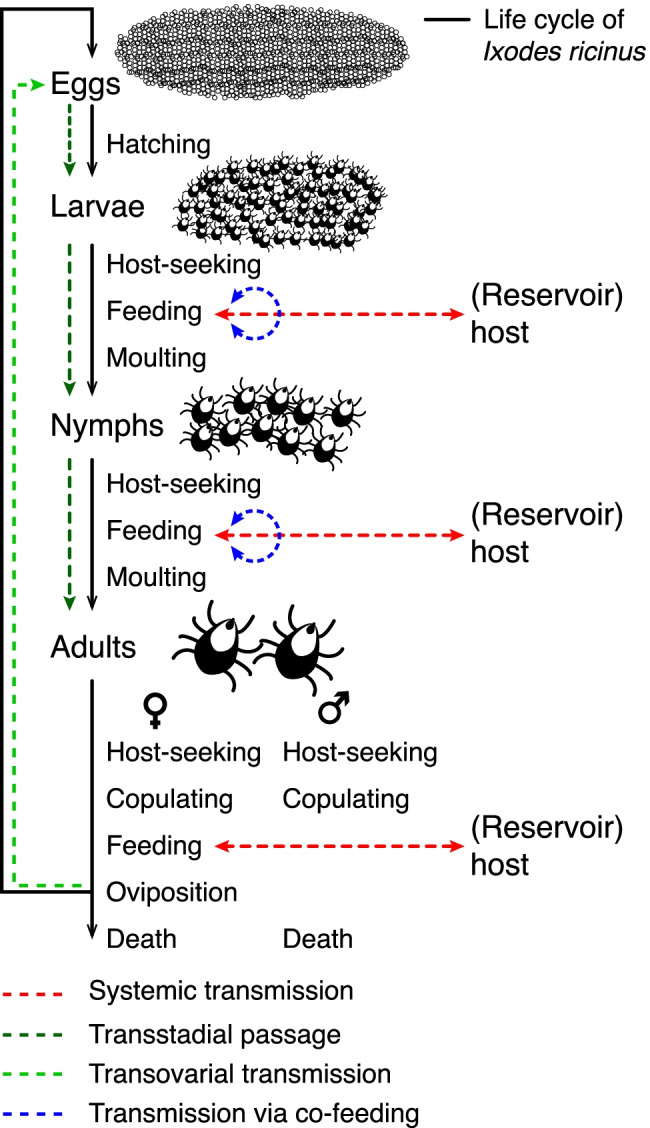
The life cycle of *Ixodes ricinus*. In general, the number of ticks in each stage is about one order of magnitude smaller than in the preceding stage (Randolph 1998). Approximately 200 larvae hatch from 2000 eggs, which molt after successful blood meals into 20 nymphs and then after further blood meals into 2 adults (female and male). Additionally, the *Borrelia burgdorferi* s.l. cycle between *I. ricinus* as the main vector and (reservoir) hosts are given.

The transmission cycle of *B. burgdorferi* s.l. is closely related to the life cycle of their vectors, ticks of the genus *Ixodes* (Figure 3). In Europe, the life cycle of *I. ricinus* comprises four developmental stages: egg, larva, nymph, and adult (female/male). Each of the three post-embryonic life stages (larvae, nymphs, adult females) must take a blood meal to reach the next stage or––in the case of female adults––to produce and deposit eggs (Figure 3). Generally, *I. ricinus* complete their life cycle within 3 years, but it can take up 6 years depending on environmental conditions such as weather, length of cold periods, temperature, and others all of which induce quiescence, developmental diapauses, or behavioral diapauses (Gray et al. 2016). Depending on the geographical regions and microclimatic conditions, larvae are active roughly from end of April to end of October. In northern and central Europe, nymphs and adults are active from March to November with a peak in April or May and a decrease during warm and dry periods (e.g., in summer months). During very mild weather periods in winter, nymphs and adults can also become active. Late winter onsets in spring cause temporary decreases in activity (Gray et al. 2016). Knowing the biotic and abiotic variables that influence tick density enables forecasting the next year’s tick density. In the case of nymphal *I. ricinus*, the variables are the mean annual temperature of the previous year, the current mean winter temperature, and the fructification of the European beech 2 years before (Brugger et al. 2018; Bregnard et al. 2020, 2021). The latter is representative for the different types of tree seeds that small rodents feed on. Although *I. ricinus* feeds on a broad range of vertebrate species, including rodents, birds, insectivores, reptiles, and deer, only a few species are known to act as a reservoir host for *B. burgdorferi* s.l., i.e., host species that participate significantly in the natural circulation of the bacteria (Gern et al. 1994, 1997, 1998; Kahl et al. 2002; Estrada-Peña et al. 2017; Wolcott et al. 2021). Principal reservoir hosts are rodents, such as *Apodemus sylvaticus*, *A. flavicollis*, and *Myodes glareolus* (previously *Clethrionomys glareolus*); insectivores, such as *Sorex minutus* and *Erinaceus europaeus*; hares such as *Lepus europaeus*; lizards such as *Psammodromus algirus* and *Lacerta agilis*; or bird species such as *Turdus* sp. or *Parus major* (Gern et al. 1998; Kurtenbach et al. 1998, 2002b; Dsouli et al. 2006; Lindgren and Jaenson 2006; Ekner et al. 2011; Norte et al. 2013; Heylen et al. 2014a, b, 2017; Norte et al. 2020a).

During attachment and feeding of a vector tick on a (reservoir) host, spirochetes can be transmitted from an infected tick to a host or vice versa known as systemic transmission which is the main route how ticks acquire borreliae from an infected host (Figure 3, Kurtenbach et al. 2002b; Mannelli et al. 2011). The passage of spirochetes acquired during feeding of one tick life stage through molting to the next stage(s), i.e., from larva to nymph to adult, is called transstadial passage (Gern and Humair 2002). In very rare cases, *B. burgdorferi* s.l. is also transmitted from the adult female to the next generation called transovarial transmission, but this scenario may depend on the tick and *Borrelia* species involved (Gern and Humair 2002; Lindgren and Jaenson 2006; van Duijvendijk et al. 2016). In addition, the so-called co-feeding transmission, i.e., feeding of infected and uninfected ticks in spatiotemporal proximity to each other on the same reservoir, has extensively discussed previously (reviewed by Voordouw 2015).

As with most tick-borne pathogens, *B. burgdorferi* s.l. is acquired during the larval or nymphal stage and is transmitted to new hosts mainly by nymphs or adults (Kurokawa et al 2020). Adults usually feed on non-reservoir competent hosts like deer (Jaenson and Tälleklint 1992; Mysterud et al. 2014). Nymphs are considered the main vector for *B. burgdorferi* s.l. to humans, as the number of nymphs is an order of magnitude greater than the number of adults, they are smaller, and thus more difficult to detect than adults (Randolph and Craine 1995; Hubálek 2009; Diuk-Wasser et al. 2012). Animal models indicate that *B. burgdorferi* s.l. transmission may occur as early as 16 h and frequently as early as 24 h, but this process depends on the involved host, tick, and *Borrelia* species or even individual *Borrelia* strains (Kahl et al. 1998; des Vignes et al. 2001). Nevertheless, the minimum attachment for a successful *Borrelia* transmission to humans has never been determined. Several studies dealing with the transmission of *Borrelia* to humans suggest a low risk of developing LB even after a bite of an infected tick (Fryland et al. 2011; Huegli et al. 2011; Wilhelmsson et al. 2016; Markowicz et al. 2021). In these studies, approximately 5% of bitten individuals were infected with spirochetes (established by analyzing seroconversion) and participants who developed symptoms ranged between 2 and 3%. In a study in the Rhine-Main area (Germany), antibodies against borreliae were detected in about 15% of blood-donor samples (Hunfeld et al. 1998).

Furthermore, the prevalence of *B. burgdorferi* s.l. in host-seeking *I. ricinus* can vary greatly by region and by year. Two meta-analyses were conducted for the periods 1984–2003 and 2010–2016, respectively, that showed an increasing prevalence from west to east Europe (Rauter and Hartung 2005; Strnad et al. 2017). Interestingly, the overall mean prevalence did not increase during that time: it was 13.7% for the first period and 12.3% for the second period. While the prevalence in adults was higher (18.6%) in the first period than in the second period (14.9%), it is almost the same in nymphs (10.1 % vs. 11.8%). Similarly, long-term studies, analyzing data over time periods of 6 to 10 years, have shown that in the Netherlands, infection rates of questing *I. ricinus* remained stable when the whole period of time was considered. Only in 2 years (2004, 2005) during the investigation, higher prevalences were observed which, however, declined to “normal” values afterwards (Coipan et al. 2013). A long-term study conducted in Latvia collected ticks for a time period of 12 years (1999–2010) in the same habitats. The results were surprising because they showed an initially high prevalence of *Ixodes* infected with *B. burgdorferi* s.l. followed by a steady decline of prevalences from 2001 to 2010 (Okeyo et al. 2020). The reasons for such a decline are as yet unknown.

To conclude, ixodid ticks seem to be an optimal vector for the spread and survival of *B. burgdorferi* s.l. for the following reasons:

1. Duration of the feeding period for larvae up to 4 days, nymphs 5 days, and female adults 10 days (Kahl 1989) favors transmission of microorganisms in both directions (tick to host or host to tick) if the tick is vector-competent and the host reservoir-competent.
2. Bacterial uptake and transmission take place via four routes: systemic, transstadial, transovarial, and via co-feeding, but efficiency of transmission may be strain dependent (Tonetti et al. 2015).
3. The broad host range of *Ixodes* spp. (more than 300 vertebrate species including rodents, birds, insectivores, reptiles, see Gern and Humair 2002; Estrada-Peña et al. 2017) enables a transmission between different hosts. However, it needs to be considered that not all *Borrelia* species are able to use all vertebrate hosts as a reservoir, and thus, some hosts may negatively influence transmission of certain *Borrelia* species.
4. Hosts enable a spatial dispersal of ticks through passive transport during feeding: about 200–300 m per generation through rodents (Rudenko et al. 2014), about 50–100 hectares by deer (Lindgren and Jaenson 2006), and much larger distances through migratory passerine birds (Hasle et al. 2009; Vollmer et al. 2011, 2013).
5. High reproductive potential as female adults are capable of laying up to 2,000 eggs.
6. Ability to withstand or circumvent most environmental constraints through quiescence or diapause and/or longer periods of starvation prolongs the life cycle and therefore also the infected period.

## Strategies of *B. burgdorferi* s.l. to survive in ticks and vertebrate hosts

*Borrelia burgdorferi* s.l. have developed sophisticated strategies to successfully perpetuate in their vector-host-transmission cycle (e.g., Anguita et al. 2003; Kung et al. 2013; Coburn et al. 2020). They can persistently infect and survive in their reservoir hosts for prolonged periods of time without causing any signs of a disease in the infected animals. In fact, spirochetes developed numerous strategies to overcome the innate and the adaptive immune response to prevent elimination by the host’s immune system (Hyde 2017; Kurokawa et al. 2020; Lin et al. 2020; Anderson and Brissette 2021). The ~1780 genes in the genome of *B. burgdorferi* s.s. strain B31 M1 are located on a single linear chromosome (910 kbp) and ~21 linear and circular plasmids (Fraser et al. 1997; Casjens et al. 2000; Pal and Fikrig 2003; Schwartz et al. 2021) and can be regulated to respond to environmental changes. Spirochetes are capable to alter the regulation of multiple genes involved in cell metabolism, motility, interaction with host- and tick-derived molecules, and thereby impacting responses of their vectors and hosts. The regulation of genes is primarily driven by three distinct systems: the RpoN-RpoS alternative sigma (σ) factor cascade, the Hk1(histidine kinase 1)-Rrp1 (response regulatory protein) two-component system (TCS) including the secondary messenger c-di-GMP, and the DksA (DnaK suppressor) and Rel_Bbu_ regulons (summarized in Samuels et al. 2021). *Borrelia* gene expression seems to be mainly affected by temperature, pH, nutrients (organic and short-fatty acids), osmolarity, carbon dioxide, oxygen, metals, or cell density (Stevenson et al. 1995; Carrol et al. 1999; Revel et al. 2002; Anguita et al. 2003; Pal and Fikrig 2003, reviewed in Samuels et al. 2021). The most prominent examples for differential gene regulation are OspA and OspC known to play a major role in colonization of the tick midgut and transmission to the vertebrate host and establishment of an infection (Pal and Fikrig 2003; Pal et al. 2004; Radolf et al. 2012; Kung et al. 2013; Tilly et al. 2016).

### Evading host innate immunity

Concerning the strategies used by *B. burgdorferi* s.l. to overcome innate immunity of different hosts, one molecular driver contributing to host speciation of *B. burgdorferi* s.l. appears to be strain-specific complement evasion of *Borrelia* by producing allelic variable complement-inactivating proteins (Kraiczy 2016a, b; Coburn et al. 2020; Skare and Garcia 2020; Lin et al 2020; Hart et al. 2021).

Common to all vertebrates and human, complement acts as a powerful surveillance system and, therefore, forms an important cornerstone of innate immunity to recognize, label (opsonize), and eliminate invading microorganisms. Upon recognition, complement activation finally leads to the generation of a cell-destroying, membrane attack complex (MAC) on the spirochetal surface. Noteworthy, species of the *B. burgdorferi* s.l. complex largely vary in their ability to survive in the presence of complement obtained from different vertebrates (Kurtenbach et al. 1998, 2002a; Kraiczy 2016a, b; Tufts et al. 2019; Lin et al. 2020; Sürth et al. 2021). Apparently, the ability of a *Borrelia* species to resist complement-mediated killing by a particular host’s serum almost always correlated with the capability of this specific *Borrelia* species to successfully infect and survive in that host and utilize it as reservoir (Kurtenbach et al. 2002a; Lin et al. 2020). While *Borrelia* species such as *B. afzelii*, *B. spielmanii*, *B. bavariensis*, *B. bissettiae*, *B. mayonii*, and *B. japonica* generally survive in mammalian but not in avian sera, *B. garinii* and *B. valaisiana* known to be associated with birds, resist killing by avian complement (Kurtenbach et al. 1998, 2002a; Kraiczy 2016a; Lin et al. 2020). In addition, *B. burgdorferi* s.s. frequently isolated from mammalian hosts and birds survived in these sera indicating that this *Borrelia* species is more likely to be considered a “generalist” (Lin et al. 2020). In fact, the characteristic serum resistance pattern(s) raises the possibility that complement contributes to *Borrelia* transmissibility, host adaptation, and overall dispersal of spirochetes in nature and, thus, plays an important role in *Borrelia* ecology.

### Complement-interacting proteins of Lyme borreliae

*Borrelia* are well-equipped with polymorphic immune evasion molecules affecting complement at different activation levels and that are produced at distinct times points during the tick-mammalian infection cycle to achieve an utmost protection against the harmful attack of hosts innate immunity (Bykowski et al. 2007; Kraiczy 2016b; Hart et al. 2018; Marcinkiewicz et al. 2019; Coburn et al. 2020; Lin et al. 2020; Hart et al. 2021). These allelically distinct and genetically unrelated molecules display specific complement-inhibitory activities. Due to the mode of inactivation, they are classified as proteins that indirectly inhibit complement by capturing host-derived complement regulators (C4BP, factor H (FH), factor H-like protein-1 (FHL-1), factor H-related proteins (FHR)) from the fluid phase or as proteins that directly bind to distinct complement components (C1r, C4b, C7, C8, and C9 or the formed membrane attack complex (MAC)) and thereby specifically inhibit complement activation (Kraiczy 2016b; Lin et al. 2020; Skare and Garcia 2020). The first group consists of the C4BP-binding protein, p43, the FH/FHL-1-binding proteins CspA and CspZ, and the FH/FHR-binding OspE/F-related (Erp) proteins ErpA, ErpC, and ErpP (Kraiczy et al. 2001; Pietikainen et al. 2010; Kraiczy 2016b; Hellwage et al. 2001). The FH/FHL-1/FHR-binding proteins are collectively referred to as “complement regulator-acquiring surface proteins or CRASP” (Kraiczy et al. 2001). Functional analyses revealed that recruitment of complement regulators C4BP and FH/FHL-1 result in an efficient inactivation of either C4b or C3b and consequently in the inhibition of all activation pathways. The second group includes at least two proteins (BBK32 and OspC) targeting the early activation steps of the classical (CP) and lectin pathway (LP) by interfering with C1r or C4b, and three molecules (CspA, BGA66, and BGA71) that inhibit MAC formation by binding to the late complement components C7, C8, and C9, respectively (Hallström et al. 2013; Hammerschmidt et al. 2014, 2016; Caine et al. 2017; Garcia et al. 2016).

Among complement-interacting proteins, BBK32, CspA, CspZ, BGA66, and BGA71 confer resistance to complement-mediated killing (Hallström et al. 2013; Hammerschmidt et al. 2014, 2016; Garcia et al. 2016). Moreover, recent observations support the notion of an adapted immune evasion strategy to affect complement at different time points of the tick-mammalian infection cycle. CspA protects spirochetes in the tick gut during the feeding process (Hart et al. 2018), while CspZ is produced when spirochetes enter and persist in the murine host (Marcinkiewicz et al. 2019). The expression profile of the *bbk32* gene suggests a complement protective role of BBK32 during the early stage of dissemination and at late time points when spirochetes infect tissues (Lin et al. 2015; Caine and Coburn 2015). In contrast, OspC appears to operate only at the initial phase of infection by either binding to the anti-complement tick Salp15 protein or by direct interaction with C4b, thereby preventing spirochetes from opsonophagocytosis (Ramamoorthi et al. 2005; Caine et al. 2017). Whether the polymorphic OspE proteins shield spirochetes from complement-mediated killing in vivo still remains unclear. Previous findings indicate that OspE proteins can promote spirochete dissemination and transmission from tick to the vertebrate host (Lin et al. 2012). Furthermore, the strain- and species-specific polymorphism among OspE proteins and their ability to bind FH from diverse vertebrates (Stevenson et al. 2002; Mühleip et al. 2018) raises the possibility that these molecules most likely participate in immune evasion and host tropisms of Lyme borreliae. It should be considered that almost all data collected so far have been obtained from studies investigating *B. burgdorferi* s.s. and, therefore, additional yet unidentified proteins of other *Borrelia* species may also possess anti-complement activities.

### Evading host adaptive immunity by antigenic variation

All Lyme borreliae species carry a *vls* (*vmp*-like sequence) locus consisting of a single expression site (*vlsE*) and a variable number of silent cassettes to escape from the host’s acquired immune response (Zhang et al. 1997; Norris 2014). By random segmental gene conversion (“switching”) events, portions of the variable domain of the *vlsE* expression site are replaced by homologous sequences of any of the silent cassettes to generate an antigen in which the variable region differs from the initial VlsE protein. Importantly, the switching events in *B. burgdorferi* calculated to be in theory 3.3 × 10^−2^ per cell per generation (Norris 2014; Verhey et al. 2018; Chaconas et al. 2020) do not occur during in vitro culture or in infected ticks (Zhang and Norris 1998; Indest et al. 2001). In mice, variable sequences in the VlsE protein can be detected as early as 4 days post-infection, and after 28 days, every single *Borrelia* cell isolated from infected tissues carries sequence variations in the *vlsE* genes as a result of individual recombination events (Zhang and Norris 1998). By altering the outer surface composition, in particular due to a continuous process of modifying highly immunogenic antigens like VlsE, the heterogeneous *Borrelia* population generated allows a subpopulation to be unrecognized by the host immune system and, thus, escape from the adaptive immune response. Moreover, the appearance of new VlsE variants shortly after transmission of spirochetes perfectly matches the timing when the first IgM antibodies are detectable in the host, in general at two weeks of initial infection, to eliminate the invading pathogen. In succession, the humoral immune response remains always a step behind, thus enabling *Borrelia* cells to persistently infect their reservoir hosts. Noteworthy, *B. burgdorferi* s.s. B31 lacking linear plasmid lp28-1 on which the *vls* locus is arranged at the right telomere are efficiently eliminated from immunocompetent mice within 3 weeks post-infection but could be cultivated without the pressure of an adaptive immune system from infected tissues of SCID mice deficient in T and B lymphocytes (Labandeira-Rey et al. 2003; Purser et al. 2003). These findings strongly indicate that antigenic variation is an essential requisite of Lyme borreliae to avoid immune surveillance and to maintain the natural life cycle.

## The disease: general aspects, case definitions, diagnosis, and treatment

Lyme arthritis or later on LD was first reported in the late 1970s by Steere et al. and is named after the town “Old Lyme,” Connecticut, in the USA (Johnson et al. 1984; Steere et al. 1977, 2016), while in Eurasia, the term LB is used. Since 1991, LD is a nationwide notifiable disease in the USA with 25,000–30,000 confirmed cases reported to public health systems annually (Borchers et al. 2015; CDC 2019). In 1996 and 2008, an increase of case numbers in the USA were noticed, likely due to the improvement of diagnostics (1996) and the introduction of a clearer case definitions in 2008 (Schwartz et al. 2017). It remains to be seen whether the updated guidelines by Wormser et al. (2021) will also show effects. In a more recent analysis based on case numbers in health insurance data, Kugeler et al. (2021) estimated that during 2010–2018, approximately 476,000 persons were diagnosed with LB annually (which also includes false-positive test results) in the USA. In Germany, available data suggest an incidence of LB between 60,000 and 200,000 cases annually (Müller et al. 2012; Rauer et al. 2020). Of note, incidences based on health insurance data also include false diagnoses due to the consultation of several health practitioners by the same patient or misassignment of the DRG Code, etc. (Müller et al. 2012; Kugeler et al. 2021). Thus, the lower numbers may result from underreporting while the higher numbers are overestimations and the true number of LB cases lies very likely somewhere in between (Müller et al. 2012; Hofmann et al. 2017; Rauer et al. 2020). But why is it so difficult to get accurate numbers for LB cases? The answer lies on one hand in the complexity of the system: it is demanding to get accurate data and numbers on tick densities, infection prevalences of ticks with *Borrelia*, tick bites, and other signs of infection that may go unnoticed. : On the other hand, inaccuracies with the reporting systems in spite of clear clinical case definitions and the uncertainties in diagnostic methods make it difficult to obtain accurate numbers of human LB cases (Kugeler and Eisen 2020). The gold standard of microbiological diagnostics is the cultivation of the causative agents. *Borreliae* are fastidious bacteria that need a very rich culture medium for their growth. These bacteria also grow very slowly under in vitro conditions (generation time 8–12 h) (Barbour 1984; Preac-Mursic et al. 1986; Wang et al. 2004). The number of spirochetes in human biopsies or specimens is often extremely low making adaptation of the bacteria to culture conditions very difficult because adaptation to an artificial medium is a strong selection process (Norris et al. 1997; Stupica et al. 2011). Clinical and laboratory data (often based on the detection of anti-*Borrelia* antibodies and borrelial DNA by PCR) as well as the history of a tick bite need to be taken into consideration. Moreover, the interpretation of test results requires ample diagnostic experience as well (Steere et al. 2016; Stanek et al. 2011). As already mentioned above, guidelines for diagnosis of LB (Mygland et al. 2010; Stanek et al. 2011; Hofmann et al. 2017; Gocko et al. 2019; Rauer et al. 2020) and the reporting of LB cases are highly inconsistent in European countries, especially due to different case definitions and country-specific reporting systems (Smith and Takkinen 2006; Schotthoefer and Frost 2015; Stone et al. 2017; Sykes and Makiello 2017).

In general, LB is characterized as a multisystemic disorder whose symptoms can be confused with other infections and which can be separated into early and late manifestations. Early manifestations may be localized or disseminated and may present as erythema migrans (EM), *Borrelia* lymphocytoma, Lyme neuroborreliosis, carditis, or ophthalmic borreliosis (Stanek et al. 2011; Hofmann et al. 2017; Rauer et al. 2020). Erythema migrans or bull’s-eye rash in North America is the most common objective clinical manifestation of LB reported in about 70–90% of the cases (e.g., Rauer et al. 2020). It was first described about 110 years ago by the Swedish physician Arvid Afzelius (Afzelius 1921; Steere 2006) and is defined as an expanding, reddish skin lesion directly at the biting site which occurs 3–30 days after the bite of an infected tick (Huppertz et al. 1999; Stanek et al. 2011; Hofmann et al. 2017; Hyde 2017; Stone et al. 2017; Sykes and Makiello 2017; van den Wijngaard et al. 2017). Often, LB might be accompanied by other symptoms like fever, fatigue, headache, mild stiff neck, arthralgia, or myalgia (Stanek et al. 2011; Borchers et al. 2015). Although EM may often be self-limiting, in some cases other organs such as skin, central nervous system, and joints, can be affected due to dissemination of the pathogen (Stanek et al. 2011; Rauer et al. 2020). This may result in more severe symptoms such as mono- and oligoarthritis of large joints, meningoradiculoneuritis (Garin-Bujadoux-Bannwarth syndrome) often combined with facial palsy, or in very rare cases (4% of patients with Lyme neuroborreliosis) myelitis with spastic atactic gait disturbance and bladder dysfunction as an affect of the disease to central nervous system (Stanek et al. 2011; Hyde 2017; Rauer et al. 2020). Based on studies from Sweden and Germany, EM was diagnosed in 77% (Sweden) and 89% (Germany) of LB cases, while Lyme neuroborreliosis was observed in 16% (Sweden) and 3% (Germany). Lyme arthritis was diagnosed in 7% (Sweden) and 5% (Germany) of LB cases (Berglund et al. 1995; Huppertz et al. 1999). The clinical presentation of the disease is at least in part similar between Europe and North America. Due to the different *Borrelia* species, clinical manifestations like borrelial lymphocytoma (early disseminated manifestations), late neuroborreliosis, or acrodermatitis chronica atrophicans (both late manifestation) are mainly reported from Europe (Glatz et al. 2015; Steere et al. 2016). The diagnosis of LB is highly based on the presence of specific clinical symptoms, but confirmation by reliable laboratory diagnostics, except for EM, is strongly recommended (Lindgren and Jaenson 2006; Stanek et al. 2011; Leeflang et al. 2016; Hofmann et al. 2017; Petrulionienė et al. 2020; Rauer et al. 2020). As many LB patients do not recall a tick bite or an EM, serological testing of anti-*Borrelia* antibodies is the mainstay of laboratory diagnostic. Direct detection of spirochetes in different specimens often lack sufficient specificity or sensitivity, e.g., by culturing of spirochetes and PCR-based methods, although the latter have been improved in recent years. In cases of acute Lyme neuroborreliosis, the chemokine CXCL13 shows significantly elevated levels, and ELISA tests provided very promising results for diagnostic utility (Rupprecht et al. 2018). By reviewing 16 different guidelines from Europe and North America for the diagnosis of LB, Eldin et al. (2019) concluded that a two-tier serology should be the method of choice consisting of an initial sensitive screening test (generally ELISA) which—in the case of a positive test result—should be followed by a confirmatory test such as an immunoblot (Strle and Stanek 2009; Dessau et al. 2018; Lohr et al. 2018; Mead et al. 2019). According to the duration of clinical symptoms and manifestations, a guideline-based, 14–21-day course of antibiotic therapy is recommended to eradicate the pathogen (Hansmann 2009; Steere et al. 2016; Gocko et al. 2019; Rauer et al. 2020). Unfortunately, there is no vaccine against human LB on the market; therefore, personal protection as wearing long clothes, using tick repellents, and checking the body for ticks after outdoor activity is an important prophylaxis (Schotthoefer and Frost 2015; Petrulionienė et al. 2020; Lantos et al. 2021)

## Epidemiological data: an ongoing challenge

As already mentioned, there are considerable uncertainties in epidemiological data, especially regarding LB incidence and prevalence (Lindgren and Jaenson 2006; Hofmann et al. 2017; Rauer et al. 2020).

Reports based on seroprevalence did not find an increase in the overall incidence (Semenza and Menne 2009; Vanthomme et al. 2012; Medlock et al. 2013; Cuellar et al. 2019; Woudenberg et al. 2020). In fact, a retrospective study conducted in Finland on the *Borrelia* seroprevalence of the population from 1962 to 1972 revealed a seroprevalence of 20% which is much higher than that reported for 2011 (3.9%) (Cuellar et al. 2019). Seemingly, the situation in Europe is similar to that in the USA, where from 2008 to 2015 annual cases of LD are almost stable in high incidence regions where *Borrelia*-infected *I. scapularis* ticks have been endemic, while cases have increased in neighboring countries (Schwartz et al. 2017). Also, the annual number of reported human LB cases per 100,000 inhabitants varies greatly across Europe and within individual countries (from 0.6 in Ireland to 300 in Austria) (Lindgren and Jaenson 2006). Germany is a typical example of how challenging it is to get reliable and comparable data of LB cases. As discussed by Wilking and Stark (2014) and Enkelmann et al. (2018), there is no nationwide mandatory notification system for LB cases. Currently, physicians and/or laboratories in nine of the 16 German federal states are obliged to report LB cases to their local health authorities. As shown by Enkelmann et al. (2018), peaks in case numbers are clearly correlated to the changes in reporting. The same situation holds true for the USA (Schwartz et al. 2017).

To address the drawback of an inconsistent reporting procedure, the European Commission added Lyme neuroborreliosis to the list of diseases for epidemiological surveillance in 2018. All EU member states are obliged to submit their available national data to the European Centre for Disease Prevention and Control (ECDC) to establish a uniform case definition but also treatment and reporting system. However, the member states are not yet obliged to introduce a new mandatory notification (EU 2018/945, The Lancet 2018). Comparing LB incidences, tick density data, and *Borrelia* prevalence data across Europe would be an important step to better assess the risk of acquiring a *Borrelia* infection and developing LB. Implementation of appropriate measures such as adequate training and education of health professionals about the topic will advance diagnostic certainty. Also, specific tick control programs may help leveling out risk hot spots and thus would sensitize the population even more to this topic. In the case of tick-borne encephalitis (TBE), a viral human pathogen that is also transmitted by ticks, the nationwide notification has led to improve epidemiological data. Potential risk areas are validated and adapted from time to time, and specific recommendations such as vaccination (for TBE) by health professionals can be made. Standard definitions and reporting systems also produce more reliable data about the progression of infections over time, e.g., due to climate change or other factors (e.g., changes in the behavior of people going into the nature because of the coronavirus pandemic) (Hellenbrand et al. 2019; RKI 2021).

## Risk assessments for human exposure to *B. burgdorferi* s.l.

Prevention and control of LB is an important aspect for public health considerations. Therefore, the factors that regulate and trigger the epidemiological triangle of the pathogen, vector, and hosts must be clearly identified and examined (Margos et al. 2011; Lou and Wu 2017). Such factors comprise climatic parameters (e.g., temperature, precipitation, relative humidity), environmental parameters (e.g., land cover, altitude, normalized difference vegetation index (NDVI)), and host or human-related parameters (e.g., density, immune response to the pathogen, regeneration, or urban greening). Risk assessments, which quantify the spatial and/or temporal risk for humans to acquire an infection caused by *B. burgdorferi* s.l., refer either to an acarological risk, i.e., the density and distribution of host-seeking *Borrelia*-infected ticks, or directly to human incidence (Killilea et al. 2008; Margos et al. 2011). Basically, there are two methodological approaches to understand the dynamics of the complex ecosystem and to quantify the risk for humans to get infected: statistical models, which aim to describe the relationship between risk (infected ticks or human incidence) and predictors, and mathematical models, which aim to simulate the vector-host-pathogen-transmission cycle including the population dynamics of ticks (Norman et al. 2015). In North America, first models and risk assessments were presented as early as the 1980s (Norman et al. 2015) and have been continuously improved until now (Sharareh et al. 2017; Gaff et al. 2020). Unfortunately, a Europe-wide risk assessment for LB is still missing. As listed in Table 2, there are only a few risk maps for selected regions in Europe. These static risk assessments differ in the spatial resolution ranging from administrative district level to 1 km^2^ or smaller. Generally, the choice of spatial and temporal model resolution determines the degree of accuracy, realism, and general applicability of a risk assessment (Kitron 1998). Although a high spatial and temporal resolution is often desired, the lowest possible resolution is mainly determined by the resolution of the available predictor variables (e.g., temperature or other key drivers) and/or data on humans, hosts, vectors, and pathogen. In case of LB risk assessments, a map depicting the spatial distribution on a regional scale would be realistic with a spatial resolution of 1–5 km. A risk map with comparable resolution was recently published by Walter et al. (2020) for TBE, the most common viral tick-borne disease (TBD) in Europe. Nevertheless, simulations, prediction, and/or risk assessments for LB are important tools for decision-makers. Such tools can support the identification of areas constituting a risk, the implementation of streamlined pathogen control methods, or help to increase disease awareness and encourage people to take preventive actions against tick bites (Norman et al. 2015). Recent analysis concluded that especially targeted awareness-based strategies are both cost-effective and significantly reduce the number of LB cases (Sharareh et al. 2017, Behler et al. 2020).

**Table 2:** Existing maps depicting the risk of human exposure to *Borrelia burgdorferi* s.l. in European regions. The models are divided in the two approaches based either on infected ticks or on human incidence. Methods and variables are indicated. The latter are grouped into climatic variables (e.g., temperature), soil variables (e.g., land cover, normalized difference vegetation index (NDVI), topographic variables (e.g., altitude), human-related variables (e.g., human population), and host-related variables (e.g., roe deer density).

Risk map depicting	Country (region)	Method	Explanatory variables					Reference
			Climatic variables	Soil variables	Topographic variables	Human related variables	Host related variables	
Density of infected ticks	Italy (Province of Trento)	Tree-based classification model with bootstrap aggregation	-	x	x	-	x	Rizzoli et al. (2002)
Density of infected ticks	Italy (Friuli Venezia Giulia region)	Multiple regression model	x	x	x	-	x	Altobelli et al. (2008)
Probability of the presence of infected ticks	Ireland	Random forests model	x	x	x	-	-	Zintl et al. (2020)
Density of infected ticks	United Kingdom (Scotland)	Mechanistic, agent-based model	x	x	-	-	x	Li et al. (2016)
Human incidence	Belgium	Negative binomial regression model	-	x	-	x	x	Linard et al. (2007)
Human incidence	Belgium	Regression trees model	-	x	-	x	-	Barrios et al. (2013)
Relative risk for human Lyme borreliosis	Czech Republic (Central Bohemian region)	Geographical information system (GIS)	-	-	-	x	-	Zeman (1997)

## Factors that might contribute in an increase of LB cases

The risk to become infected with Lyme borreliae largely depends on contact of humans with vectors infected with the pathogen (Borchers et al. 2015). If the vector, potential reservoir hosts, and the human population expand their range, it is strongly suggested that LB will be an important public health issue in the future (de Keukeleire et al. 2016; Stone et al. 2017; Petrulionienė et al. 2020). What are the key drivers of distribution expansion and an increasing infection risk for humans? Encroachment of humans into natural habitats is one factor that increases the risk of infection. As known from other VBD, the vector-host-pathogen transmission cycle is a highly complex ecosystem in which ticks, *Borrelia*, and vertebrate hosts interact at various environmental conditions with each other. Consequently, each change driven either by natural phenomena or by human interventions directly affects the ecological balance and thus the potential of the pathogen to come into contact with humans (Patz et al. 2000; Hartemink et al. 2015; Estrada-Peña et al. 2017). Anthropogenic impacts such as climate change, land cover changes, or geographical expansion of hosts (also incidental or dead-end hosts such as humans) are considered to be the main reasons for the increase in tick abundance, distribution of ticks in new habitats, and therefore the risk of infection (Mysterud et al. 2014; Li et al. 2019; Diuk-Wasser et al. 2020). As both, *Ixodes* ticks as well as *Borrelia*, depend on complex ecological systems, one needs to ask the question why are *Ixodes* abundance and *Borrelia* prevalence are increasing when there is a general consent that most ecosystems are in a dire condition (Wagner et al. 2021)? Different species may respond differently to environmental changes and some may even increase (at least temporarily). Insect decline can be taken as a proxy for the general decline of ecosystems. Several reservoir hosts such as hedgehogs or other insectivores depend on insects as major food source, and thus, if these hosts disappear as a consequence of insect decline, it may have a knock-on effect on *Borrelia* and may lead to a reduction or shifts in *Borrelia* species composition. For some European bird populations, stable or increasing numbers have been recorded while others appear to dwindle (Staneva and Burfield 2017). How these biotic and abiotic factors may impact tick ecology and expansion is currently difficult to assess.

### Impact of climate change on tick ecology

Ticks are poikilothermic organisms; thus, rising temperatures have an enormous impact on all stages of the tick’s life cycle and their distribution as well as on their associated microorganisms and pathogens and hosts (Perez et al. 2016; Alkishe et al. 2017). Within the last decades, climate change led not only to an increased annual mean temperature of about 0.7°C; it is expected to rise an additional 1.1°C within the next 100 years (Patz et al. 2000). Climate change also results in more extreme weather events (e.g., local flooding, droughts, storms) (EPA 2021) with an unknown effect on eco-systems.

These rising temperatures greatly affect the phenology of ticks as they might extend their seasonal activity and feeding behavior in spring and fall in some regions while in other regions conditions may become too hot and dry (Alkishe et al. 2017; Petrulionienė et al. 2020). It is assumed that *I. ricinus* could prolong their usual activity period from March to November until January due to milder winters (Gray 2008; Gray et al. 2009; Porretta et al. 2013). Besides that, a climatic shift to milder winters can lead to an expanding of the tick’s distribution to both, northern latitudes and higher altitudes (Semenza and Menne 2009; Korotkov et al. 2015; Semenza and Suk 2018; Bouchard et al. 2019). A study from the Czech Republic showed that *I. ricinus* had shifted their distribution level from about 700 to 800 m to 1,100 m.a.s.l over the last 2 decades, and in Switzerland, ticks can be found at altitudes up to 1,450 m.a.s.l. (Cotty et al 1986; Materna et al. 2008). Studies from Sweden and Norway reported also a shift of the northern distribution level of *I. ricinus* (Jaenson et al. 2012; Jore et al. 2014). On the other hand, changes in temperature and moisture due to climate change may have an impact on woodlands, trees, or other natural habitats that are important for the hosts. Such changes may have a yet unpredicted outcome on the ecology in various regions in Europe. In addition, lower summer precipitation combined with higher summer temperatures as suggested for the southernmost distribution range of *I. ricinus* might have a negative effect on the survival of this tick species and thus decreases the risk of infection (Gray et al. 2009; Porretta et al. 2013). Furthermore, higher temperatures might also improve the tick’s and/or host survival and reproduction conditions resulting in higher tick and/or host densities, possible leading to more infected ticks and/or hosts (Mills et al. 2010). Another important factor for the development of ticks is humidity as they need up to 80% relative humidity to survive during the off-host period (Gray et al. 2009; Medlock et al. 2013). Changes in temperature impacts the hydrological cycle as warmer air can hold more moisture than cooler air, resulting in more precipitation events providing suitable humidity conditions for the tick’s survival. However, this can also result in reduced soil moisture in case of more evaporation which might affect ticks negatively (Patz et al. 2000). Another effect of expanding milder temperatures is the extension of vegetation periods which also affects the abundance of ticks and hosts as well as the pathogen (Menzel and Fabian 1999; Jaenson and Lindgren 2011). As shown in Sweden, milder climate favors the production of plant biomass in particular deciduous vegetation which might have a positive effect on mammals, roe deer, or birds known to serve as hosts for *I. ricinus* (Jaenson et al. 2009; Jaenson and Lindgren 2011).

It is also assumed that the development and replication of a pathogen within an ectothermic vector is faster at higher temperatures, and therefore, the risk of transmission may increase (Caminade et al. 2019; Bouchard et al. 2019). On the other hand, there are physiological constraints for the tick as molting from one stage to the next requires times for diapauses which cannot be shortened.

### Conservation management

Ticks are restricted to habitats with moderate to high precipitation and an adequate vegetation cover that holds humidity during dry periods (Lindgren et al. 2000; Gray et al. 2009; Medlock et al. 2013). Therefore, vegetation characteristics are very important for the tick’s distribution and survival (Lindgren and Jaenson 2006). Additionally, the local composition of potential hosts as well as their abundance influences the number of infected ticks (Medlock et al. 2013; Rudenko et al. 2014). Thus, conservation management activities affecting land use by, e.g., woodland regeneration or urban greening as well as wildlife management strategies to control invasive and native host populations will influence the dynamic system of VBD (Millins et al. 2017). Another worrying development from an ecological perspective is fragmentation of landscapes as these splits animals into small and isolated populations making them vulnerable to species losses which may have uncertain consequences on VBD systems. Moreover, species characterized by high population densities and being active at a low range like small rodents could benefit from the lack of predators or competitors. Previous studies in the USA and elsewhere showed that fragmented forests smaller than 2 ha tend to harbor high densities of white-footed mice combined with low densities of large vertebrate hosts (e.g., deer) resulting in a high density of infected nymphal ticks (Patz et al. 2000; Allan et al. 2003). In such cases, woodland regeneration could result in a higher biodiversity and thus in a significant reduction of *Borrelia*-infected ticks (Younger 2016). Another factor which may influence the potential risk of acquiring *Borrelia* is ongoing urban greening with the creation and establishment of protected and recreational areas as it could increase the likelihood of human-tick contact (Millins et al. 2017). However, ongoing urbanization and increases in artificial land cover provide poor settings for wildlife that can serve as hosts for ticks (European Environment Agency (EEA) 2011).

Wildlife fluctuation, either human made or naturally induced, also influences the distribution of hosts and, thus, affects tick abundance and infection risk (de Keukeleire et al. 2016). Although deer are known to act as dead-end hosts for *Borrelia*, they are an important host for adult female ticks laying eggs afterward (Perkins et al. 2006; Millins et al. 2017; Hofmeester et al. 2017). Changes in land use and climate as well as the expansion of non-native deer species have led, in some parts of the world, to an expansion of the range and population size of deer. (Newson et al. 2012). Because natural predators have decreased or are completely absent due to human’s activity, in many countries, deer are generally controlled by culling or fencing for forestry and crop protection, but there is also a growing interest in using these strategies to control tick populations and thus the incidence of TBD (Gilbert et al. 2012). Studies examining the effects of deer control on the tick population revealed that the reduction of the deer population through culling or fencing also reduced the incidence of ticks, but in some cases, it led to an increased number of ticks in small areas of exclusion (e.g., Gilbert et al. 2012; Perkins et al. 2006; Hofmeester et al. 2017). Beside deer, smaller vertebrate hosts such as the invasive Siberian chipmunk (*Tamias sibiricus barberi*) should be controlled because of the high prevalence of *Borrelia*-infected animals that might enhance the infection rate of feeding ticks more than native rodent species (Marsot et al. 2013; Millins et al. 2017; Mori et al. 2018). Other aspects of wildlife management like winter feeding of deer seems to play a tangential role as the benefits of these measures for deer are not clear (Petersen and Messmer 2011).

### Changes in human behavior: outdoor activity and self-protection

Beside biotic and abiotic factors affecting vectors and reservoir hosts, an additional aspect leading to a higher risk for humans to get infected with LB includes the frequency of contact between humans and infected ticks (Jaenson et al. 2012). Due to milder temperatures in Europe, the time humans spend on outdoor or recreational activities (e.g., forestry works, berry and mushroom picking, biking, and hiking) have increased. People who are exposed to ticks during outdoor activity have a higher risk to become infected; thus, it has been recommended that wearing long clothes and using tick repellents will reduce the risk of tick bites (Petrulionienė et al. 2020). As ticks feed for several days on their host, the time of attachment could be also important for the personal risk assessment. Mice infection studies showed that transmission of *Borrelia* occurs not before 24h but there are no reliable data for humans so far (Piesman 1993; Hojgaard et al. 2008). Thus, an increased awareness of getting “attacked” by ticks and inspecting the body for attached ticks could prevent transmission of the pathogens (Kahl et al. 1998; des Vignes et al. 2001).

## Conclusion

Lyme Borreliosis is the most prevalent VBD in the Northern Hemisphere, but the current knowledge about the complex interaction between diverse pathogen (*B. burgdorferi* s.l. complex) and vectors (*I. ricinus, I. persulcatus*) as well as different reservoir and non-reservoir hosts is still incomplete. Additionally, epidemiological data for all European countries are hitherto largely fragmented, and inconsistent reporting systems and case definitions make a European-wide evaluation of LB difficult. Due to anthropogenic-driven environmental changes, the occurrence of ticks increases in many areas in Europe although seroprevalence studies do not support an increase in exposure of the human population to *Borrelia*. Therefore, to significantly increase scientific knowledge, it is essential to more diligently scrutinize the biology of this complex eco-epidemiological system and human LB case numbers. Data about the actual burden of this disease are undoubtedly needed for health authorities to implement and improve surveillance and control measures, in particular in the light that no vaccine is currently available.

## Data Availability

Not applicable
